# Supplementary material for: Tunable genetic devices through simultaneous control of transcription and translation
Source: Nat Commun. 2020 Apr 29;11:2095. doi: 10.1038/s41467-020-15653-7 (PMC7190835; doi:10.1038/s41467-020-15653-7)
Supplement: Supplementary file 1 — Supplementary Information [file 41467_2020_15653_MOESM1_ESM.pdf]

Supplementary Information for:

Tunable genetic devices through simultaneous control of transcription and translation

Vittorio Bartoli, Grace A. Meaker, Mario di Bernardo and Thomas E. Gorochowski

**Supplementary Notes**	**Page**
Supplementary Note 1: Modelling the tunable expression system	2
Supplementary Note 2: Modelling possible retroactivity effects	4
**Supplementary Figures**	
Supplementary Figure 1: Characterization of sensor systems	6
Supplementary Figure 2: Simulated response functions for models	7
Supplementary Figure 3: Plasmid maps	8
Supplementary Figure 4: Flow cytometry automated gating approach	9
**Supplementary Tables**	
Supplementary Table 1: Modelling parameters	10
Supplementary Table 2: Sequences used for thermodynamic modeling	11
Supplementary Table 3: Performance summary of tunable NOR gate	12
Supplementary Table 4: Synthesized DNA fragments used in this study	13
Supplementary Table 5: Primers used in this study	14
**Supplementary References**	15

Supplementary Note 1: Modelling the tunable expression system

To better understand the behavior of the tunable expression system (TES), we derived the following set of pseudo-chemical reaction equations to capture changes in the concentrations of the four main species in our system: toehold switch transcript *S*, tuner sRNA *T*, switch-sRNA complex *C*, and output protein *P* (see **Figure 1a** for a schematic of the device)

$$\xrightarrow{\alpha_T+\alpha_T^0} T \ , \quad (1)$$

$$\xrightarrow{\alpha_S+\alpha_S^0} S \ , \quad (2)$$

$$T + S \xrightarrow{k_{C+}} C \ , \quad (3)$$

$$C \xrightarrow{k_{C-}} T + S \ , \quad (4)$$

$$C \xrightarrow{\alpha_P} C + P \ , \quad (5)$$

$$P \xrightarrow{\gamma_P} \emptyset \ , \quad (6)$$

$$T \xrightarrow{\gamma_T} \emptyset \ , \quad (7)$$

$$S \xrightarrow{\gamma_S} \emptyset \ , \quad (8)$$

$$C \xrightarrow{\gamma_C} \emptyset \ . \quad (9)$$

Here, α_S and α_T are the induced promoter activities (rate of transcript production) of the main and tuner inputs, respectively, and α_P is the production rate of the output protein. α_S^0 and α_T^0 are the basal rates of transcription of the main and the tuner input promoters, respectively. k_{C+} and k_{C-} are the binding and unbinding rates of the toehold switch transcript with tuner sRNA. γ_S, γ_T, γ_C, γ_P are first-order degradation rates of the main toehold switch transcript, tuner sRNA, switch-sRNA complex, and output protein, respectively. Stochastic simulations of these chemical reaction equations were performed using COPASI (**Methods**) and a Systems Biology Markup Language (SBML) file capturing the model is available in **Supplementary Data 1**.

From these chemical reactions, we derived a set of ordinary differential equations (ODEs) to model the dynamics of the TES and assess how its function is affected by key design parameters. The model tracks changes in the concentrations of the main toehold switch transcript *S*, tuner sRNA *T*, switch-sRNA complex *C*, and output protein *P*. It consists of the following four coupled ODEs that capture production and loss of each species due to complex formation or degradation

$$\frac{dS}{dt} = \alpha_s^0 + \alpha_S - k_{C+}ST + k_{C-}C - \gamma_S S \ , \quad (10)$$

$$\frac{dT}{dt} = \alpha_T^0 + \alpha_T - k_{C+}ST + k_{C-}C - \gamma_T T \ , \quad (11)$$

$$\frac{dC}{dt} = k_{C+}ST - k_{C-}C - \gamma_C \, C, \quad (12)$$

$$\frac{dP}{dt} = \alpha_P C - \gamma_P P. \tag{13}$$

All species and rates match those in the chemical reactions captured by **Equations (1)–(9)**. To analyze the behavior of this system, numerical simulations of these ODEs were performed using Python (**Methods**) across a range of biologically realistic parameter values (**Figure 3**; **Supplementary Table 1**).

Supplementary Note 2: Modelling possible retroactivity effects

One way that retroactivity [1, 2] could arise in the TES is due to the demand that synthesis of an output protein (e.g. YFP) places on the shared ribosome pool of a cell [3]. If expression of the output protein is sufficiently burdensome, there would be significant drop in the free ribosomes available to synthesize core machinery such as RNA polymerase (RNAP) and thereby a reduction in the transcription rates of the TES. To better understand this effect, we modified our existing model (**Supplementary Note 1**) to incorporate a factor r_e that captures the relative availability of host resources (i.e. RNAP)

$$\frac{dS}{dt} = (\alpha_S^0 + \alpha_S)r_e - k_{C+}ST + k_{C-}C - \gamma_S S\,, \tag{14}$$

$$\frac{dT}{dt} = (\alpha_T^0 + \alpha_T)r_e - k_{C+}ST + k_{C-}C - \gamma_T T\,, \tag{15}$$

$$\frac{dC}{dt} = k_{C+}ST - k_{C-}C - \gamma_C\, C. \tag{16}$$

To calculate r_e we coupled these equations to a delay differential equation (DDE) model of ribosome allocation dynamics [3]. This model considers a fixed total pool of ribosomes R_t and their allocation between endogenous transcripts E (modelled as a single "average" type, see [3] for details), translationally active TES complexes C, and a free ribosome pool R_f. Conservation of the total number of ribosomes per cell is ensured by the additional algebraic constraint

$$R_f = R_t - [R_E + R_C(t)], \tag{17}$$

where R_E and R_C represent allocation of ribosomes to endogenous transcripts and active TES complexes, respectively, whose changes are captured by the following equations

$$\frac{dR_E}{dt} = \alpha_E R_f(t)E - \alpha_E R_f(t - \tau_E)E, \tag{18}$$

$$\frac{dR_C}{dt} = \alpha_C R_f(t)C(t) - \alpha_C R_f(t - \tau_C)C(t - \tau_C). \tag{19}$$

Here, α_E and α_C are the translation initiation rate of the endogenous transcripts and active TES complexes, respectively. Each equation captures the initiation of free ribosomes onto a respective transcript and the removal of ribosome from a transcript back into the free ribosome pool after the specific translation time of the transcript, either τ_E for endogenous transcripts or τ_C for active TES complexes. The relative availability of core cellular machinery like RNAP is then given by

$$r_e = \frac{R_E}{R_0}, \tag{20}$$

where R_0 is the steady state value of R_E in the model when only endogenous genes are present (i.e. no TES complexes are considered, C = 0).

Analysis of the model was performed by numerical simulation of the DDEs above using Julia (**Methods**) across a range of biologically realistic parameter values (**Supplementary Table 1**). We then considered two cases: 1. when TES transcript synthesis rate was coupled

to ribosome availability (as above), and 2. when there was no coupling between the output protein production rate and the synthesis rate of the TES transcripts (i.e. r_e = 1). The simulations showed that retroactivity (case 1) could impact the response function of the TES device, but only when the output caused a significant burden on the cell and only for the most highly expressed outputs, i.e., when both the input and tuner promoter activities were high (**Supplementary Figure 2**).

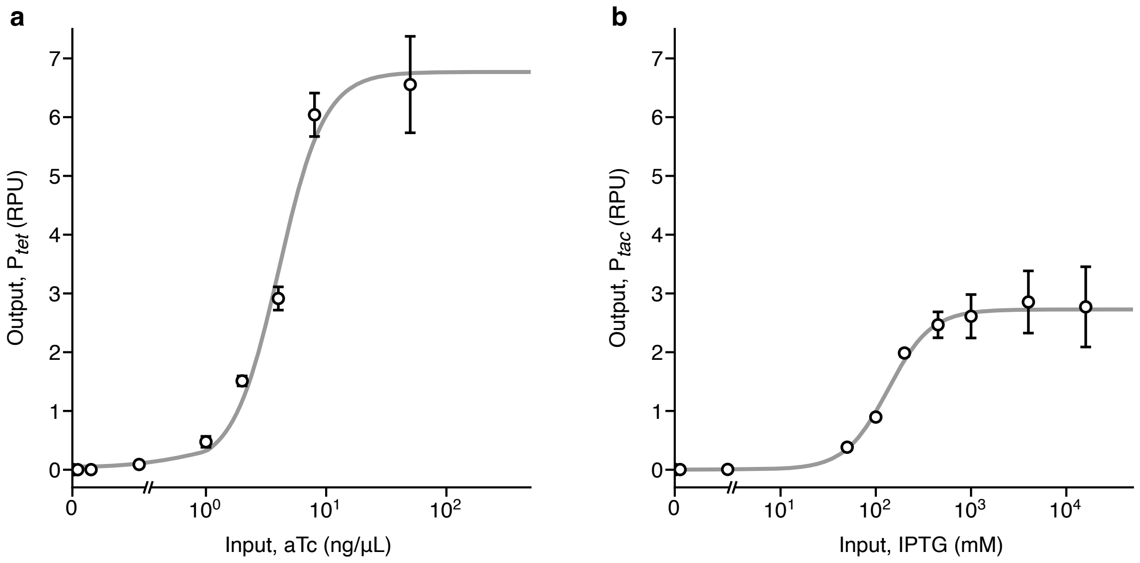

Supplementary Figure 1: Characterization of sensor systems. (**a**) Response function of the P_{tet} promoter to varying aTc concentrations: 0, 0.01, 0.04, 0.14, 0.5, 1, 2, 4, 8, 50 ng/μL. (**b**) Response function of the P_{tac} promoter to varying IPTG concentrations: 0, 0.5, 5, 50, 100, 200, 450, 1000, 4000, 16000 mM. Points denote average output in relative promoter units (RPU) (**Methods**) from three biological replicates with error bars showing ±1 standard deviation. Solid grey lines show fitted Hill functions (P_{tet} fit parameters: y_{min} = 0.05 RPU, y_{max} = 6.77 RPU, K = 4.0 ng/μL aTc, n = 2.3; P_{tac} fit parameters: y_{min} = 0.001 RPU, y_{max} = 2.73 RPU, K = 134 mM IPTG, n = 1.9).

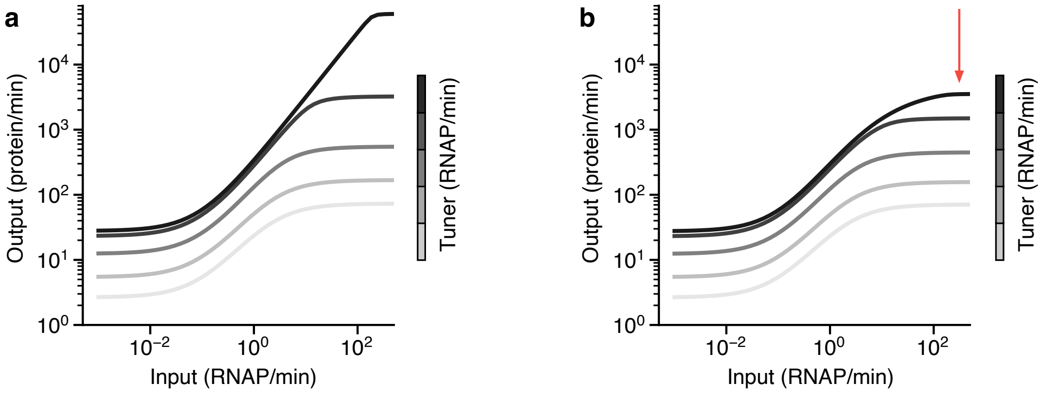

Supplementary Figure 2: Simulated response functions for models. (**a**) Response functions when TES transcription is not coupled to varying availability of core cellular resources (r_e = 1). (**b**) Response functions when TES transcription is influenced by varying availability of core cellular resources due to synthesis of the output protein. Red arrow shows the decrease in the TES protein output due to retroactivity. All plots show individual curves for tuner promoter activities: 0.0001, 0.3, 1.5, 10, 190 transcripts/min (light grey to black). Model described in **Supplementary Note 2**.

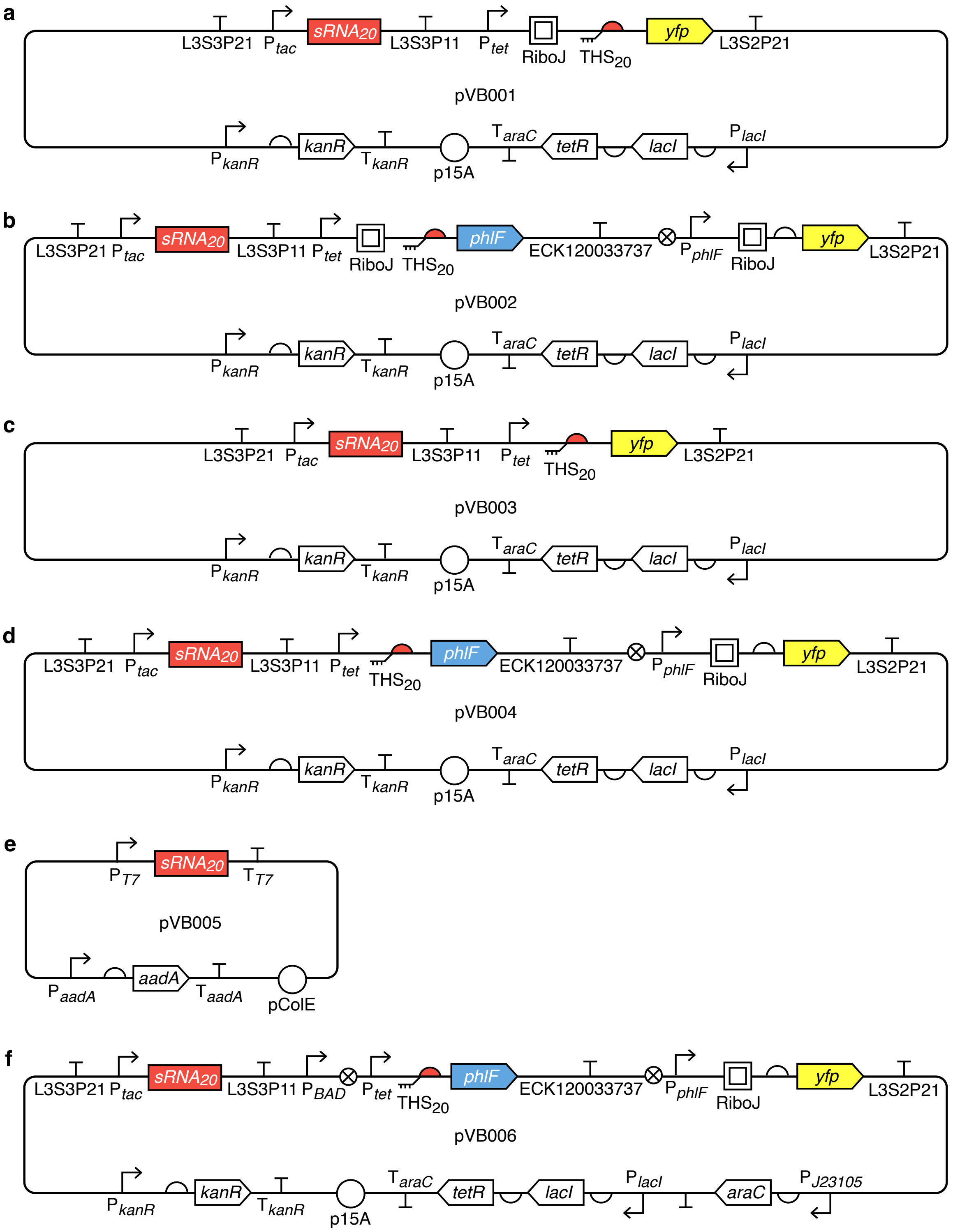

Supplementary Figure 3: Plasmid maps. (**a**) pVB001: Tunable expression system. (**b**) pVB002: Tunable genetic NOT gate. (**c**) pVB003: Tunable expression system without RiboJ insulating element. (**d**) pVB004: Tunable genetic NOT gate without RiboJ insulating element. (**e**) pVB005: Tuner sRNA booster. (**f**) pVB006: Tunable NOR gate.

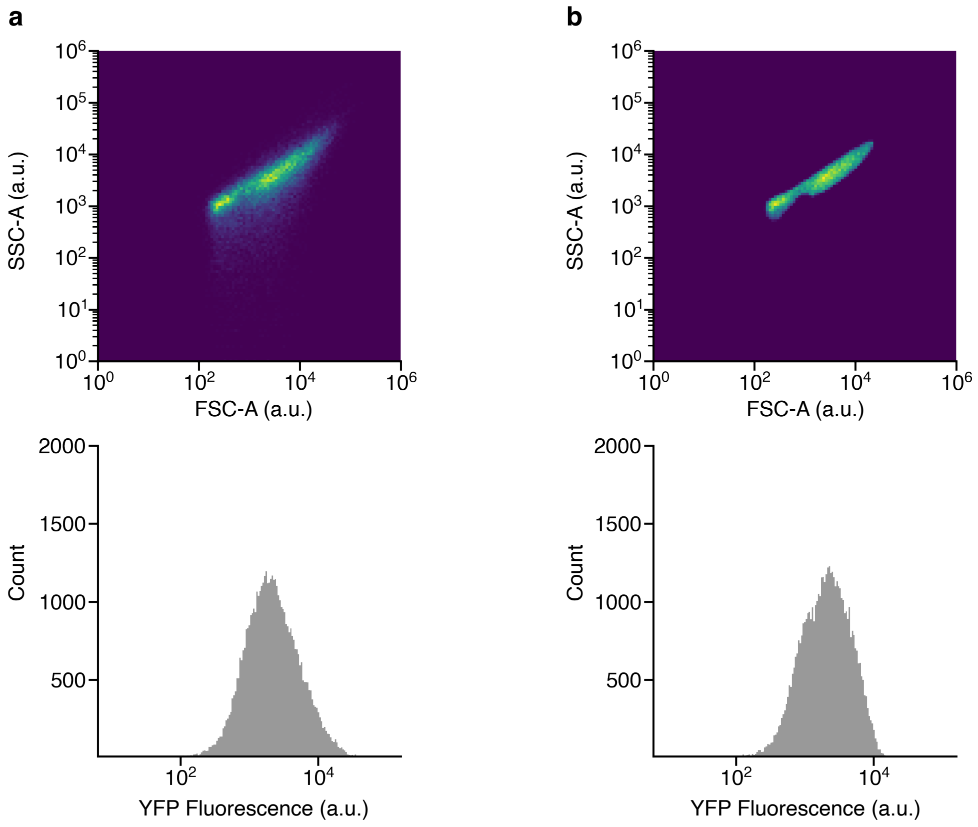

Supplementary Figure 4: Flow cytometry automated gating approach. (**a**) Raw event data from the flow cytometer. (**b**) Automatically gated event data with unwanted debris removed (**Methods**). Gating is performed using FlowCal which implements density-based filtering to discard events that deviate in their appearance from the main population. Top pseudo color density scatter plots show the forward and side scatter area (FSC-A and SSC-A) and bottom histogram shows the associated raw YFP fluorescence from these events. Data is collected for the initial TES construct with RiboJ present and an input of 6.6 RPU and tuner of 2.6 RPU.

Supplementary Table 1: Modelling parameters.

Name	Description	Value(s)	Units	Refs.
α_S	Induced transcription rate of toehold switch promoter	0–300[a]	transcripts min^{-1}	[4,5,6]
α_T	Induced transcription rate of tuner sRNA promoter	0–190[b] or 730[c]	transcripts min^{-1}	[4,5,6]
α_S^0	Basal transcription rate of toehold switch promoter	0.0886[d]	transcripts min^{-1}	[4,5,6]
α_{T^0}	Basal transcription rate of tuner sRNA promoter	0.2307 or 0.9228[d]	transcripts min^{-1}	[4,5,6]
α_P	Protein synthesis rate from toehold switch	5	proteins $complex^{-1}$ min^{-1}	[7]
k_{C+}	Association rate of switch and trigger RNAs into complex	0.0257[e]	complexes $transcript^{-1}$ min^{-1}	[8]
k_{C-}	Dissociation rate of switch and trigger RNAs from complex	0.00672[e]	transcripts $complex^{-1}$ min^{-1}	[8]
γ_S	Degradation rate of toehold switch transcript	0.231[f]	min^{-1}	[9]
γ_T	Degradation rate of trigger sRNA	0.231[f]	min^{-1}	[9]
γ_C	Degradation rate of toehold switch and sRNA complex	0.231[f]	min^{-1}	[9]
γ_P	Degradation rate of output protein	0.035[g]	min^{-1}	[10]
R_t	Total number of ribosomes	26300	ribosomes	[3]
E	Number of endogenous transcripts	4140	transcripts	[3]
α_E	Translation initiation rate of endogenous transcripts	0.0002	$ribosome^{-1}min^{-1}$	[3]
α_C	Translation initiation rate of active TES complexes	0.0034	$ribosome^{-1}min^{-1}$	[3]
τ_E	Translation time of average endogenous gene	0.28	min	[3]
τ_C	Translation time of TES output gene	5.67	min	[3]

a. We assume that the induced translation initiation rate of P_{tet} is similar to the transcription rate of an average constitutively expressed endogenous gene (20 transcripts min^{-1}) [2] and that a plasmid with a p15A origin of replication has 15 copies per cell [3].
b. The induced transcription initiation rate of P_{tet} is 1.57 times higher than P_{tac} [1].
c. We assume the pColE1 origin of replication leads to 50 copies per cell [7] and thus the transcription initiation rate from all P_{tac} promoters is ~4 times higher when the sRNA booster is present.
d. Uninduced basal expression calculated based on fold-change activation measured in previous work [1] and the estimated induced transcription initiation rates (α_S, α_T).
e. We assume the rates of DNA hybridization are similar to RNA hybridization [5].
f. We assume the half-life of the switch transcript and tuner sRNA are similar to the average mRNA half-life in a cell, measured to be 3 min in exponentially growing cells [6].
g. Dilution by growth is the dominant mode of degradation. The doubling time of cells during these experiments was determined to be ~20 min.

Supplementary Table 2: Sequences used for thermodynamic modeling.

Name	RNA sequence
Toehold Switch	GGGCGUUAAUCUCUGGCUUGCUUUAUGUCUGUAAACAGAGGAGAUACAGAAUGAAAGCAAGCAACCUGGCG GCAGCGCAAAAGAUGCGUAAA
Tuner sRNA	GGGUCAUGACUGGGACACGCCAGUCAUGAGAAUACAGACAUAAAGCAAGCCAGAGAUUAACGAAG
Cleaved Toehold Switch with RiboJ	UCACCGGAUGUGCUUUCCGGUCUGAUGAGUCCGUGAGGACGAAACAGCCUCUACAAAUAAUUUUGUUUAAG GGCGUUAAUCUCUGGCUUGCUUUAUGUCUGUAAACAGAGGAGAUACAGAAUGAAAGCAAGCAACCUGGCGG CAGCGCAAAAGAUGCGUAAA
Full Toehold Switch with RiboJ	AGCUGUCACCGGAUGUGCUUUCCGGUCUGAUGAGUCCGUGAGGACGAAACAGCCUCUACAAAUAAUUUUGU UUAAGGGCGUUAAUCUCUGGCUUGCUUUAUGUCUGUAAACAGAGGAGAUACAGAAUGAAAGCAAGCAACCU GGCGGCAGCGCAAAAGAUGCGUAAA

Supplementary Table 3: Performance summary of tunable NOR gate.

Tuner (RPU)	**Dynamic range**[a] (a.u.) Ara / aTc			**Fold-change**[a] Ara / aTc			**Intersection**[a] Ara / aTc		
	+ / –	– / +	+ / +	+ / –	– / +	+ / +	+ / –	– / +	+ / +
0.002	3210 ± 573	2623 ± 322	3671 ± 233	5.6 ± 3.9	4.3 ± 2.6	16.7 ± 3.3	0.21 ± 0.12	0.13 ± 0.07	0.04 ± 0.003
2.6	3808 ± 604	4013 ± 718	4012 ± 729	8.4 ± 0.1	15.3 ± 4.5	13.9 ± 4.5	0.07 ± 0.04	0.08 ± 0.02	0.05 ± 0.02

a. Measurements are a comparison to experiments when both input inducers (Ara and aTc) are absent. Average values are shown ± 1 standard deviation calculated from flow cytometry data for three biological replicates.

Supplementary Table 4: Synthesized DNA fragments used in this study.

Name	Sequence
TES-P1	CGCAAACCGCCTCTCCCCGCAACGATCGTTGGCTGTGTTGACAATTAATCATCGGCTCGTATAATGTG<br>TGGAATTGTGAGCGCTCACAATTGGGACCGTGGACCGCATGAGGTCCACGGTAAACATAACTATAACA<br>AGCCTACAATTCATTCAAACCCAATTATTGAACACCCTTCGGGGTGTTTTTTTGTTTCTGGTCTACCT<br>ACTCCACCGTTGGCTTTTTTCCCTATCAGTGATAGAGATTGACATCCCTATCAGTGATAGAGATAATG<br>AGCACAGCTGTCACCGGATGTGCTTTCCGGTCTGATGAGTCCGTGAGGACGAAACAGCCTCTACAAAT<br>AATTTTGTTTAAGGGCGTTAATCTCTGGCTTGCTTTATGTCTGTAAACAGAGGAGATACAGAATGAAA<br>GCAAGCAACCTGGCGGCAGCGCAAAAGATGCGTAAACGCAGCCTGAATGGCGAATG
TES-P2	CGCAAACCGCCTCTCCCCGCAACGATCGTTGGCTGTGTTGACAATTAATCATCGGCTCGTATAATGTG<br>TGGAATTGTGAGCGCTCACAATTGGGTCATGACTGGGACACGCCAGTCATGAGAATACAGACATAAAG<br>CAAGCCAGAGATTAACGAAGCCAATTATTGAACACCCTTCGGGGTGTTTTTTTGTTTCTGGTCTACCT<br>ACTCCACCGTTGGCTTTTTTCCCTATCAGTGATAGAGATTGACATCCCTATCAGTGATAGAGATAATG<br>AGCACAGCTGTCACCGGATGTGCTTTCCGGTCTGATGAGTCCGTGAGGACGAAACAGCCTCTACAAAT<br>AATTTTGTTTAAGGGTGAATGAATTGTAGGCTTGTTATAGTTATGAACAGAGGAGACATAACATGAAC<br>AAGCCTAACCTGGCGGCAGCGCAAAAGATGCGTAAACGCAGCCTGAATGGCGAATG
NOR-P1	TTAGGCGGCCGCAACGATCGTTGGCTGTGTTGACAATTAATCATCGGCTCGTATAATGTGTGGAATTG<br>TGAGCGCTCACAATTGGGTCATGACTGGGACACGCCAGTCATGAGAATACAGACATAAAGCAAGCCAG<br>AGATTAACGAAGCCAATTATTGAACACCCTTCGGGGTGTTTTTTTGTTTCTGGTCTACCACTTTTCAT<br>ACTCCCGCCATTCAGAGAAGAAACCAATTGTCCATATTGCATCAGACATTGCCGTCACTGCGTCTTTT<br>ACTGGCTCTTCTCGCTAACCAAACCGGTAACCCCGCTTATTAAAAGCATTCTGTAACAAAGCGGGACC<br>AAAGCCATGACAAAAACGCGTAACAAAAGTGTCTATAATCACGGCAGAAAAGTCCACATTGATTATTT<br>GCACGGCGTCACACTTTGCTATGCCATAGCATTTTTATCCATAAGATTAGCGGATCCGAGACCTTAC
NOR-P2	TTACGGTCTCGGATCCTACCTGACGCTTTTTATCGCAACTCTCTACTGTTTCTCCATACCCGTTTTTT<br>TGGGCTAGCTACTCCACCGTTGGCTTTTTTCCCTATCAGTGATAGAGATTGACATCCCTATCAGTGAT<br>AGAGATAATGAGCACAGCTGTCACCGGATGTGCTTTCCGGTCTGATGAGTCCGTGAGGACGAAACAGC<br>CTCTACAAATAATTTTGTTTAAGGGCGTTAATCTCTGGCTTGCTTTATGTCTGTAAACAGAGGAGATA<br>CAGAATGAAAGCAAGCAACCTGGCGGCAGCGCAAAAGATGCGTAAAGCACGTACCCCGAGCCGTAGCA<br>GCATTGGTAGCCTGCGTAGTCCGCATACCCATAAAGCAATTCTGACCAGCACCATTGAAATCCTGAAA<br>GAATGTGGTTATAGCGGTCTGAGCATTGAAAGCGTTGCACGTCGTGCCGGTGCAAGCAAACCGACCAT<br>TTATCGTTGGTGGACCAATAAAGCAGCACTGATTGCCGAAGTGTATGAAAATGAAAGCGAACAGGTGC<br>GTAAATTTCCGGATCTGGGTAGCTTTAAAGCCGATCTGGATTTTCTGCTGCGTAATCTGTGGAAAGTT<br>TGGCGTGAAACCATTTGTGGTGAAGCATTTCGTTGTGTTATTGCAGAAGCACAGCTGGACCCTGCAAC<br>CCTGACCCAGCTGAAAGATCAGTTTATGGAACGTCGTCGTGAGATGCCGAAAAAACTGGTTGAAAATG<br>CCATTAGCAATGGTGAACTGCCGAAAGATACCAATCGTGAACTGCTGCTGGATATGATTTTTGGTTTT<br>TGTTGGTATCGCCTGCTGACCGAACAGCTGACCGTTGAACAGGATATTGAAGAATTTACCTTCCTGCT<br>GATTAATGGTGTTTGTCCGGGTACACAGCGTTAAGGAAACACAGAAAAAAGCCCGCACCTGACTGAGA<br>CCTTAC
NOR-P3	TTACGGTCTCATGACAGTGCGGGCTTTTTTTTTCGACCAAAGGTGTCAACGTTCGACGTACGGTGGAA<br>TCTGATTCGTTACCAATTGACATGATACGAAACGTACCGTATCGTTAAGGTCAAGCTGTCACCGGATG<br>TGCTTTCCGGTCTGATGAGTCCGTGAGGACGAAACAGCCTCTACAAATAATTTTGTTTAATACTAGAG<br>AAAGAGGGGAAATACTAGATGGTGAGCAAGGGCGAGGAGCTGTTCACCGGGGTGGTGCCCATCCTGGT<br>CGAGCTGGACGGCGACGTAAACGGCCACAAGTTCAGCGTGTCCGGCGAGGGCGAGGGCGATGCCACCT<br>ACGGCAAGCTGACCCTGAAGTTCATCTGCACCACAGGCAAGCTGCCCGTGCCCTGGCCCACCCTCGTG<br>ACCACCTTCGGCTACGGCCTGCAATGCTTCGCCCGCTACCCCGACCACATGAAGCTGCACGACTTCTT<br>CAAGTCCGCCATGCCCGAAGGCTACGTCCAGGAGCGCACCATCTTCTTCAAGGACGACGGCAACTACA<br>AGACCCGCGCCGAGGTGAAGTTCGAGGGCGACACCCTGGTGAACCGCATCGAGCTGAAGGGCATCGAC<br>TTCAAGGAGGACGGCAACATCCTGGGGCACAAGCTGGAGTACAACTACAACAGCCACAACGTCTATAT<br>CATGGCCGACAAGCAGAAGAACGGCATCAAGGTGAACTTCAAGATCCGCCACAACATCGAGGACGGCA<br>GCGTGCAGCTCGCCGACCACTACCAGCAGAACACCCCAATCGGCGACGGCCCCGTGCTGCTGCCCGAC<br>AACCACTACCTTAGCTACCAGTCCGCCCTGAGCAAAGACCCCAACGAGAAGCGCGATCACATGGTCCT<br>GCTGGAGTTCGTGACCGCCGCCGGGATCACTCTCGGCATGGACGAGCTGTACAAGTAAGAATTCTTAG

Supplementary Table 5: Primers used in this study.

Primer	Template[a]	Sequence[b]
F_TES_1	TES-P1	ctaacggggggcctttttttgAACGATCGTTGGCTGTGTTG
R_TES_2	TES-P1	aaagccaacggtggagtaGGTAGACCAGAAACAAAAAAACACCC
F_TES_3	TES-P2	gtgtttttttgtttctggtctaccTACTCCACCGTTGGCTTTTTTCC
R_TES_4	TES-P3	tcctcgcccttgctcacTTTACGCATCTTTTGCGCTGC
R_TES_5	pAN1720	caacacagccaacgatcgttCAAAAAAAGGCCCCCCGTTAG
F_TES_6	pAN1720	cagcgcaaaagatgcgtaaaGTGAGCAAGGGCGAGGA
F_NOT_1	pVB001	ttcgttttggtccAGTTTACGGCTAGCTCAGTCCTAG
R_NOT_2	pVB001	gctacggctcggggtacgtgcTTTACGCATCTTTTGCGC
F_NOT_3	pAN3938	tgcgtaaagcacgtaccccgaGCCGTAGCAGCATTGGTAG
R_NOT_4	pAN3938	ttccaccgtacgtcgaaCGTTGACACCTTTGGTCG
F_NOT_5	pAN4036	caaaggtgtcaacgTTCGACGTACGGTGGAATC
R_NOT_6	pAN4036	atccggtgacagcttGACCTTAACGATACGGTACGTTTC
F_NOT_7	pAN4036	taccgtatcgttaaggtcAAGCTGTCACCGGATGTG
R_NOT_8	pAN4036	tgagctagccgtaaactGGACCAAAACGAAAAAGGC
F_RiboJ_Rem	pVB001, pVB002	cgaaggtctcaGGGCGTTAATCTCTGGCTTGCTTTATG
R_RiboJ_Rem	pVB001, pVB002	ggtcggtctcagcccGTGCTCATTATCTCTATCACTGATAGGGATGTC
F_pAN1720_EcoRI	pAN1720	TTAGAATTCCATGGACGAGCTGTACAAG
R_pAN1720_NotI	pAN1720	TTAGCGGCCGCGGCGGGAGTATGAAAAGTAAG

a. Plasmid names prefixed with pAN are from Neilsen et al. [11].
b. Underlined lowercase sequences denote homology regions added for Gibson assembly.

Supplementary References

[1] Del Vecchio D., Ninfa A.J. & Sontag E.D. (2008) Modular cell biology: retroactivity and indulation. *Molecular Systems Biology* **4**, 161.

[2] Del Vecchio D. & Sontag E.D. (2009) Engineering Principles in Bio-molecular Systems: From Retroactivity to Modularity. *European Journal of Control* **3**, 389–397.

[3] Gorochowski T.E., Avcilar-Kucukgoze I., Bovenberg R.A.L., Roubos J.A. & Ignatova Z. (2016) A minimal model of ribosome allocation dynamics captures trade-offs in expression between endogenous and synthetic genes. *ACS Synthetic Biology* **5**, 710–720.

[4] Stanton B.C., Nielsen A.A.K., Tamsir A., Clancy K., Peterson T. & Voigt C.A. (2014) Genomic mining of prokaryotic repressors for orthogonal logic gates. *Nature Chemical Biology* **10**, 99–105.

[5] Kennell D. & Riezman H. (1977) Transcription and translation initiation frequencies of the *Escherichia coli lac* operon. *Journal of Molecular Biology* **114**, 1–21.

[6] Hiszczyńska-Sawicka E. & Kur J. (1997) Effect of *Escherichia coli* IHF Mutations on Plasmid p15A Copy Number. *Plasmid* **38**, 174–179.

[7] Pai A. & You L. (2009) Optimal tuning of bacterial sensing potential. *Molecular Systems Biology* **5**, 286.

[8] Xu S., Zhan J., Man, B., Jiang, S. Yue W., Gao S., Guo C., Liu H., Li Z., Wang J. & Zhou Y. (2017) Real-time reliable determination of binding kinetics of DNA hybridization using a multi-channel graphene biosensor. *Nature Communications* **8**, 14902.

[9] Bernstein J.A., Khodursky A.B., Lin P.-H., Lin-Chao S. & Cohen S.N. (2002) Global analysis of mRNA decay and abundance in *Escherichia coli* at single-gene resolution using two-color fluorescent DNA microarrays. *Proceedings of the National Academy of Sciences USA* **99**, 9697–9702.

[10] Liang S.-T., Ehrenberg M., Dennis P. & Bremer H. (1999) Decay of *rplN* and *lacZ* mRNA in *Escherichia coli*. *Journal of Molecular Biology* **288**, 521–538.

[11] Nielsen, A. A. K. *et al.* (2016) Genetic circuit design automation. *Science* **352**, aac7341.
