# Supplementary material for: Tunable genetic devices through simultaneous control of transcription and translation
Source: Nat Commun. 2020 Apr 29;11:2095. doi: 10.1038/s41467-020-15653-7 (PMC7190835; doi:10.1038/s41467-020-15653-7)
Supplement: Supplementary file 5 — Description of Additional Supplementary Files [file 41467_2020_15653_MOESM5_ESM.pdf]

Title: Supplementary Data 1:
Description: Model of the TES in Systems Biology Markup Language (SBML) format.

Title: Supplementary Data 2:
Description: Annotated sequences for all plasmids in GenBank format.
